# Generalizations of incompressible and compressible Navier–Stokes equations to fractional time and multi-fractional space

**DOI:** 10.1038/s41598-022-20911-3

**Published:** 2022-11-11

**Authors:** M. Levent Kavvas, Ali Ercan

**Affiliations:** 1 Hydrologic Research Laboratory and J. Amorocho Hydraulics Laboratory, Department of Civil and Environmental Engineering, University of California, Davis, CA 95616 USA; 2 J. Amorocho Hydraulics Laboratory, Department of Civil and Environmental Engineering, University of California, Davis, CA 95616 USA; 3 Now at Hydraulics Laboratory, Department of Civil Engineering, Middle East Technical University, Ankara, Turkey

**Keywords:** Civil engineering, Climate sciences, Hydrology, Applied mathematics

## Abstract

This study develops the governing equations of unsteady multi-dimensional incompressible and compressible flow in fractional time and multi-fractional space. When their fractional powers in time and in multi-fractional space are specified to unit integer values, the developed fractional equations of continuity and momentum for incompressible and compressible fluid flow reduce to the classical Navier–Stokes equations. As such, these fractional governing equations for fluid flow may be interpreted as generalizations of the classical Navier–Stokes equations. The derived governing equations of fluid flow in fractional differentiation framework herein are nonlocal in time and space. Therefore, they can quantify the effects of initial and boundary conditions better than the classical Navier–Stokes equations. For the frictionless flow conditions, the corresponding fractional governing equations were also developed as a special case of the fractional governing equations of incompressible flow. When their derivative fractional powers are specified to unit integers, these equations are shown to reduce to the classical Euler equations. The numerical simulations are also performed to investigate the merits of the proposed fractional governing equations. It is shown that the developed equations are capable of simulating anomalous sub- and super-diffusion due to their nonlocal behavior in time and space.

## Introduction

The origin of fractional differentiation goes back to a letter between Leibniz and l’Hôpital in late seventeenth century. Later, Euler^1^, Lagrange^2^, Liouville^3^, Grünwald^4^, and Riemann^5^ made significant contributions to fractional calculus^6^. Until recently, fractional calculus has been considered as a mathematical theory without real-life applications. However, starting with the last quarter of twentieth century, the fractional differentiation found numerous applications in science, mainly due to its nonlocal nature (see the next section). Such applications are found in rheology, electrochemistry, chemical physics, finance, bioengineering, continuum mechanics, image and signal processing, plasma physics, diffusion and advection phenomena^6–10^.

Navier Stokes equations (NSE) are the origin of the governing equations of flow and transport. Evidence of fractional flow and transport behavior in various fields of science was already reported, for example in hydrology and hydraulics^11–15^, anomalous transport of solutes in porous media^16–19^, and climate science^20–23^. Recently, a number of models in applied mathematics was also reported based on fractional derivatives, for example, to model propagation of long waves by fractional Korteweg-de Vries equation^24–26^, and to model diffusion by fractional Burgers’ equation^27,28^.

Expressing the conservation of mass and momentum, the NSE govern the motion of fluids in above mentioned subfields of science including flows and turbulence in the atmosphere, rivers, lakes and soil. Fractional flow and transport models showed superiority in describing anomalous diffusion^9,29,30^, intermittent turbulence^31^, chaos-induced turbulence diffusion^32^, and multifractal behavior of velocity fields of turbulent fluids at low viscosity^33^. As such, fractional Navier–Stokes equations have been generalized by researchers in the last decades by time fractional NSE (tfNSE) and/or space fractional NSE (sfNSE). Here, we generalize the governing equations of unsteady multi-dimensional incompressible and compressible NSE to fractional time and multi-fractional space. When the fractional powers in time and in multi-fractional space are unit integer values, the developed fractional equations of continuity and momentum for incompressible and compressible fluid flow reduce to the classical NSE. The derived fractional governing equations are nonlocal in time and space. As such, they can quantify the effects of initial and boundary conditions better than the classical integer order NSE.

In general, tfNSE were simply obtained by replacing the time derivative in the moment equation with a fractional time derivative^34–36^ and sfNSE were obtained by replacing the Laplacian operator by a fractional Laplacian operator^37,38^. Wu^39^ studied the existence and uniqueness of solutions of sfNSE. Starting with sfNSE, Xu et al.^38^ numerically investigated the pressure-driven flow between two parallel plates by the finite difference method. The tfNSE have been studied extensively by development of analytical solutions to special flow cases^34–36^ and numerical methods^40,41^. Carvalho-Neto and Planas^42^ explored the existence, uniqueness, decay, and regularity properties of mild solutions to tfNSE and pointed out that the time fractional derivative has affected not only the regularity in the time variable of the solution but also that in the space variable. Zhou and Peng^43^ studied the existence and uniqueness of global and local mild solutions of tfNSE. Replacing the time derivative in the moment equation with the fractional derivative in Caputo sense, El-Shahed and Salem^34^ obtained exact solutions for three special configurations. Momani and Odibat^35^ applied the Adomian decomposition method to solve the unsteady flow of a viscous fluid in a tube in which the velocity field is a function of one space coordinate. Kumar et al.^36^ studied the same problem by coupling Adomian decomposition method and Laplace transform method. However, analytical solutions of tfNSE are only available under certain assumptions about the state of the fluid and for simple configurations for the flow pattern^35^. Utilizing fractional integrals to consider the fractality of a homogeneous fractional flow medium (with a constant fractal dimension D < 3), the fractional generalization of Navier–Stokes and Euler equations were introduced by the seminal work of Tarasov^44^, in which final forms of the fractional governing equations with space fractional derivatives but integer order time derivative were developed. As such, the scaling factors appear only for space fractional derivative terms in the fractional governing equations in Tarasov^44^.

This study introduces dimensionally consistent governing equations for the motion of fluids in fractional time and multifractional space. Unlike most of the previously introduced fractional fluid flow equations, the proposed governing equations herein have scaling factors for both the fractional time and fractional space derivative terms, which also assure dimensional consistency. As explained in Zaslavsky^30^, fractional governing equations account for the anomalous flow processes, including sub-diffusive (i.e., slow) and super-diffusive (i.e., fast) processes. Numerical simulations are performed in this study to show the importance of fractional time and space derivative powers and to investigate the anomalous behaviour in the fNSE.

### Physical framework of the fractional derivative

The fractional derivative of order $0 < \alpha$ for a function f(t) in Caputo framework is defined as follows^8^

1 $${}_{{\text{a}}} D_{t}^{\alpha } f\left( t \right) = \frac{1}{{\Gamma \left( {m - \alpha } \right)}}\mathop \int \limits_{{\text{a}}}^{t} \frac{{f^{\left( m \right)} \left( \tau \right)d\tau }}{{\left( {t - \tau } \right)^{\alpha + 1 - m} }},\;\;0 \le m - 1 < \alpha < m,\;t \ge a$$

where Γ( . ) is the gamma function, and m = 1,2,3,…. Accordingly, the fractional derivative of a function depends on its values over an entire interval [a, t] and therefore can handle nonlocal effects. The fractional derivative definition in Eq. (1) can represent the fractional derivative with respect to time or space, depending on whether the variable t is defined as time or spatial location.

For $0 < \alpha \le 1$, a first-order approximation of Caputo’s fractional time derivative over a given time interval [0, T], which is divided into N equal subintervals of increment dt = T/N by using nodes t_n_ = n.dt, n = 0,1,2,…,N, can be written as^45^:

2 $$D_{t}^{\alpha } f^{n} = \frac{1}{{\Gamma \left( {2 - \alpha } \right)}}\frac{1}{{dt^{\alpha } }}\mathop \sum \limits_{j = 1}^{n} w_{j}^{\left( \alpha \right)} \left( {f^{n - j + 1} - f^{n - j} } \right)$$

where $f^{n} = f\left( {t_{n} } \right)$ and the weight ${ }w_{j}^{\left( \alpha \right)} = j^{1 - \alpha } - \left( {j - 1} \right)^{1 - \alpha }$.

**Figure 1:**
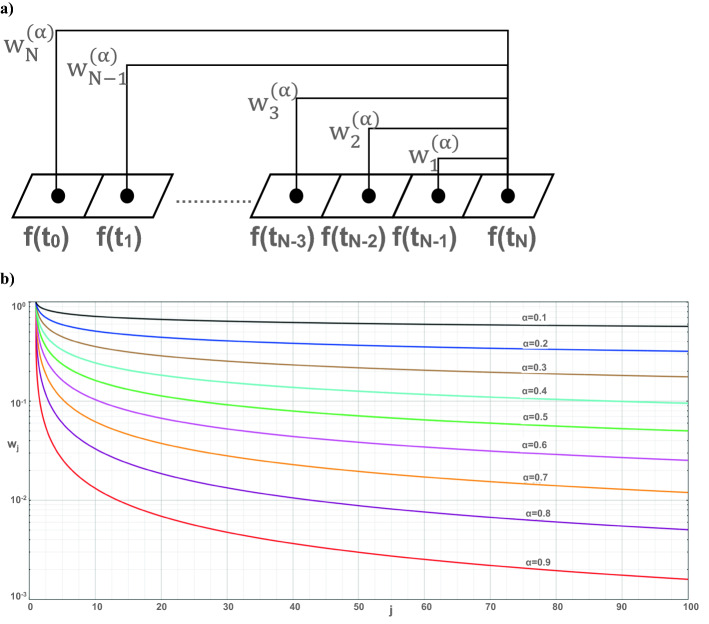
(**a**) Schematic description of weights ${w}_{j}^{\left(\alpha \right)}$ (for j = 1, 2, …, N) in estimating the fractional derivative ${D}_{t}^{\alpha }f\left({t}_{N}\right)$ and (**b**) weights as a function of j.

As shown in Eq. (2), the fractional derivative of $f\left( {t_{N} } \right)$ is estimated from the difference between $f\left( {t_{N} } \right)$ and $f\left( {t_{N - 1} } \right)$, $f\left( {t_{N - 2} } \right) \ldots$, $f\left( {t_{1} } \right),{\text{ and}}$ $f\left( {t_{0} } \right)$ with weights ${ }w_{j}^{\left( \alpha \right)}$ for j = 1, 2,…,N. The difference between $f\left( {t_{N} } \right)$ and $f\left( {t_{N - 1} } \right)$ contributes with ${ }w_{1}^{\left( \alpha \right)}$, between $f\left( {t_{N} } \right)$ and $f\left( {t_{N - 2} } \right)$ contributes with ${ }w_{2}^{\left( \alpha \right)}$, and so on (see Fig. 1a). For increasing j, weights decrease slower as $\alpha$ gets smaller, and weights decrease faster as α gets larger (see Fig. 1b). As such, function values away from $t_{N}$ contribute to the fractional derivative of $f\left( {t_{N} } \right)$ with higher weights as fractional parameter $\alpha$ decreases from 1. In other words, the effect of past lessens for time fractional derivative and the effect of long distances reduces for space fractional derivative as fractional parameter goes to 1. Similarly, the effect of past for time fractional derivative and the effect of long distances for space fractional derivative grow as fractional parameter decreases from 1. By such a definition of the fractional derivative, a physical phenomenon taking long memory in time and long-range dependence in space can be realistically modeled. Time dependence not only at the time of interest but also at previous times can be modeled by time fractional governing equations. Similarly, long-range dependence in space can be modeled by space fractional governing equations, so that the space dependence not only at the location of interest but also at other spatial locations can be realistically modeled by space fractional governing equations. As such, the fractional governing equations can consider the effect of the initial conditions for long times, and the effect of the boundary conditions for long distances.

### Continuity equation of unsteady fluid flow in fractional time and multifractional space

To $\beta$-order the Caputo fractional derivative $_a{D}_{x}^{\beta }f\left(x\right)$ of a function f(x) may be defined as^46–49^:

3 $$_{a} D_{x}^{\beta } f\left( x \right) = \frac{1}{{\Gamma \left( {1 - \beta } \right)}}\mathop \int \limits_{a}^{x} \frac{{f{}^\backprime (\xi )}}{{\left( {x - \xi } \right)^{\beta } }}d\xi ,~\;\;~~0{\text{}} < ~\beta < 1,\;\;~~x \ge a$$

where $\xi$ represents a dummy variable in the equation.

It was shown that one can obtain a $$\beta_{i}$$-order (i = 1,2,3) approximation to a function $$f\left( {x_{i} } \right)$$ around $$x_{i}$$$$- \Delta x_{i}$$ as^15^:

4 $$f\left( {x_{i} } \right) = f\left( {x_{i} - \Delta x_{i} } \right) + ~\frac{{\left( {\Delta x_{i} } \right)^{{\beta _{i} }} }}{{\Gamma \left( {\beta _{i} + 1} \right)}}~_{{x_{i} - \Delta x_{i} }} D_{{x_{i} ~}}^{{\beta _{i} }} f\left( {x_{i} } \right),\;{\text{i}} = {\text{1}},{\text{2}},{\text{3}}.$$

In Eq. (4), for *f(*$$x_{i}$$*)* =  $$x_{i}$$ an analytical relationship between $$\Delta x_{i}$$ and $$\left( {\Delta x_{i} } \right)^{\beta }$$ (i = 1,2,3$$)$$ that will be applicable throughout the modelling domain is possible when the lower limit in the above Caputo derivative in Eq. (4) is taken as zero (that is, $$\Delta x_{i}$$ = $$x_{i}$$)^15^.

**Figure 2:**
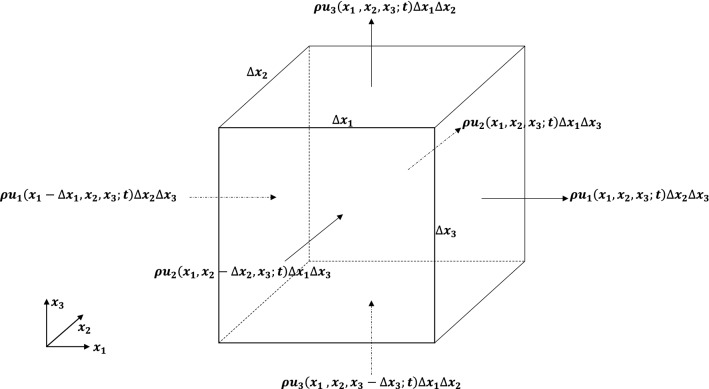
The control volume for the water flow in three-dimensional space.

Within the above framework one can express the net mass outflow rate from the control volume in Fig. 2 as:

5 $$\begin{gathered} \left[ {\rho u_{1} \left( {x_{1} ~,x_{2} ,~x_{3} ;t} \right) - \rho u_{1} \left( {x_{1} - \Delta x_{1} ~,x_{2} ,~x_{3} ;t} \right)} \right]\Delta x_{2} \Delta x_{3} + \left[ {\rho u_{2} \left( {x_{1} ~,x_{2} ,~x_{3} ;t} \right) - \rho u_{2} \left( {x_{1} ~,x_{2} - \Delta x_{2} ,~x_{3} ;t} \right)} \right]\Delta x_{1} \Delta x_{3} \hfill \\ \;\; + \left[ {\rho u_{3} \left( {x_{1} ~,x_{2} ,~x_{3} ;t} \right) - \rho u_{3} \left( {x_{1} ~,x_{2} ,~x_{3} - \Delta x_{3} ;t} \right)} \right]\Delta x_{1} \Delta x_{2} . \hfill \\ \end{gathered}$$

Then by introducing Eq. (4) into Eq. (5) with $\Delta x_{j}$ = $x_{j}$, j = 1,2,3, and expressing the resulting Caputo derivative $_{0} D_{x}^{\beta } f\left( x \right)~$ (taking $\Delta {\text{x}}$ = x renders the lower limit in the Caputo derivative of Eq. (4) to be 0) by $\frac{{\partial^{\beta } f\left( x \right)}}{{\left( {\partial x} \right)^{\beta } }}$ for convenience, the net mass flux from the control volume in Fig. 2 may be expressed to $\beta_{i}$-order, i = 1,2,3 as:

6 $$\begin{aligned} \frac{{\left( {\Delta x_{1} } \right)^{{\beta _{1} }} }}{{\Gamma \left( {\beta _{1} + 1} \right)}}\left( {\frac{\partial }{{\partial x_{1} }}} \right)^{{\beta _{1} }} \left( {\rho u_{1} \left( {\bar{x};t} \right)} \right)\Delta x_{2} \Delta x_{3} & + \frac{{\left( {\Delta x_{2} } \right)^{{\beta _{2} }} }}{{\Gamma \left( {\beta _{2} + 1} \right)}}\left( {\frac{\partial }{{\partial x_{2} }}} \right)^{{\beta _{2} }} \left( {\rho u_{2} \left( {\bar{x};t} \right)} \right)\Delta x_{1} \Delta x_{3} \\ & + \frac{{\left( {\Delta x_{3} } \right)^{{\beta _{3} }} }}{{\Gamma \left( {\beta _{3} + 1} \right)}}\left( {\frac{\partial }{{\partial x_{3} }}} \right)^{{\beta _{3} }} \left( {\rho u_{3} \left( {\bar{x};t} \right)} \right)\Delta x_{1} \Delta x_{2} \\ \end{aligned}$$

where $\overline{x} = \left( {x_{1} , x_{2} ,x_{3} } \right),$ $\rho$ is the fluid density and $u_{i} \left( {\overline{x};t} \right)$ is the component of the flow velocity vector in $x_{i}$ direction, i = 1,2,3. 

It also follows from Eq. (4) with *f(x*_*i*_*)* = *x*_*i*_ that^15^

7 $$\left( {\Delta x_{i} } \right)^{{\beta_{i} }} = \frac{{\Gamma \left( {\beta_{i} + 1} \right)\Gamma \left( {2 - \beta_{i} } \right)}}{{x_{i}^{{1 - \beta_{i} }} }} \left( {\Delta x_{i} } \right),\;\,\,{\text{i}} = {1},{2},{3}$$

with respect to $\beta_{i}$-order fractional space in the i-th direction, i = 1,2,3.

Introducing Eq. (7) into Eq. (6) yields for the net mass outflow rate through the control volume

8 $$\begin{aligned} & \frac{{\Gamma \left( {2 - \beta_{1} } \right)}}{{x_{1}^{{1 - \beta_{1} }} }} \left( {\frac{\partial }{{\partial x_{1} }}} \right)^{{\beta_{1} }} \left( {\rho u_{1} \left( {\overline{x};t} \right)} \right)\Delta x_{1} \Delta x_{2} \Delta x_{3} + \frac{{\Gamma \left( {2 - \beta_{2} } \right)}}{{x_{2}^{{1 - \beta_{2} }} }}\left( {\frac{\partial }{{\partial x_{2} }}} \right)^{{\beta_{2} }} \left( {\rho u_{2} \left( {\overline{x};t} \right)} \right)\Delta x_{1} \Delta x_{2} \Delta x_{3} \\ & \;\;\; + \frac{{\Gamma \left( {2 - \beta_{3} } \right)}}{{x_{3}^{{1 - \beta_{3} }} }}\left( {\frac{\partial }{{\partial x_{3} }}} \right)^{{\beta_{3} }} \left( {\rho u_{3} \left( {\overline{x};t} \right)} \right)\Delta x_{1} \Delta x_{2} \Delta x_{3} ,\;\,\,\overline{x} = \left( {x_{1} ,x_{2} , x_{3} } \right) \\ \end{aligned}$$

to $$\beta_{i}$$-order, i = 1,2,3.

The time rate of change of mass within the control volume in Fig. 2 may be expressed as

9 $$\frac{{\rho \left( {\overline{x}, t} \right) - \rho \left( {\overline{x}, t - \Delta t} \right)}}{\Delta t} \Delta x_{1} \Delta x_{2} \Delta x_{3}$$

Introducing Eq. (4), with fractional power $\beta_{i }$ replaced by $\alpha$, and x replaced by t, into Eq. (9), and expressing the resulting Caputo derivative operator with its lower limit as 0, by $\frac{{\partial^{\alpha } }}{{\left( {\partial t} \right)^{\alpha } }}$ for convenience, yields the time rate of change of mass within the control volume with respect to α-fractional time increments:

10 $$\frac{{\left( {\Delta t} \right)^{\alpha } }}{{\Delta t \Gamma \left( {\alpha + 1} \right)}} \left( {\frac{\partial }{\partial t}} \right)^{\alpha } \rho \left( {\overline{x}, t} \right)\Delta x_{1} \Delta x_{2} \Delta x_{3}$$

to $\alpha$-order. With respect to the Caputo derivative $_{0} D_{t}^{\alpha } t = \frac{{\partial ^{\alpha } t}}{{\left( {\partial t} \right)^{\alpha } }}~$:

11 $$\frac{{\partial^{\alpha } t}}{{\left( {\partial t} \right)^{\alpha } }} = \frac{{t^{1 - \alpha } }}{{\Gamma \left( {2 - \alpha } \right)}}$$

which when combined with Eq. (4) (with x replaced by t and $\beta_{i }$ replaced by $\alpha$) yields the approximation

12 $$\left( {\Delta t} \right)^{\alpha } = \frac{{\Gamma \left( {\alpha + 1} \right)\Gamma \left( {2 - \alpha } \right)}}{{t^{1 - \alpha } }} \left( {\Delta t} \right)$$

to $\alpha$-order. Introducing Eq. (12) into Eq. (10) yields for the time rate of change of mass within the control volume in Fig. 2 with respect to $\alpha$-order fractional time increments:

13 $$\frac{{\Gamma \left( {2 - \alpha } \right)}}{{t^{1 - \alpha } }} \frac{{\partial^{\alpha } \rho \left( {\overline{x}, t} \right)}}{{\left( {\partial t} \right)^{\alpha } }} \Delta x_{1} \Delta x_{2} \Delta x_{3} .$$

Since the time rate of change of mass within the control volume of Fig. 2 is inversely related to the net flux through the control volume, Eqs. (8) and (13) can be combined to yield

14 $$\frac{{\Gamma \left( {2 - \alpha } \right)}}{{t^{1 - \alpha } }} \frac{{\partial^{\alpha } \rho \left( {\overline{x}, t} \right)}}{{\left( {\partial t} \right)^{\alpha } }} = - \mathop \sum \limits_{i = 1}^{3} \frac{{\Gamma \left( {2 - \beta_{i} } \right)}}{{x_{i}^{{1 - \beta_{i} }} }} \left( {\frac{\partial }{{\partial x_{i} }}} \right)^{{\beta_{i} }} \left( {\rho \left( {\overline{x}, t} \right)u_{i} \left( {\overline{x}, t} \right)} \right),\,\,\overline{x} = \left( {x_{1} ,x_{2} , x_{3} } \right),$$

as the general continuity equation of unsteady, multidimensional fluid flow in fractional time and multi-fractional space which holds both for compressible as well as incompressible flows.

Fractional continuity Eq. (14) for fluid flow can also be written as

15 $$\frac{{\partial^{\alpha } \rho \left( {\overline{x}, t} \right)}}{{\left( {\partial t} \right)^{\alpha } }} = - \mathop \sum \limits_{i = 1}^{3} \frac{{t^{1 - \alpha } }}{{\Gamma \left( {2 - \alpha } \right)}}\frac{{\Gamma \left( {2 - \beta_{i} } \right)}}{{x_{i}^{{1 - \beta_{i} }} }} \left( {\frac{\partial }{{\partial x_{i} }}} \right)^{{\beta_{i} }} \left( {\rho \left( {\overline{x}, t} \right)u_{i} \left( {\overline{x}, t} \right)} \right),\,\,\overline{x} = \left( {x_{1} ,x_{2} , x_{3} } \right).$$

Performing a dimensional analysis on Eq. (15) results in

16 $$\frac{{M/L^{3} }}{{T^{\alpha } }} = \frac{{T^{1 - \alpha } }}{{L^{{1 - \beta_{i} }} }}\frac{1}{{L^{{\beta_{i} }} }}\frac{M}{{L^{3} }}\frac{L}{T} = \frac{{M/L^{3} }}{{T^{\alpha } }}$$

which shows the dimensional consistency of the fractional continuity equation of the fluid flow.

Podlubny^8^ has shown that for $n$−1 < $\alpha ,\beta_{i} < n$ where n is any positive integer, as $\alpha$ and $\beta_{i}$ $\to$ n, the Caputo fractional derivative of a function f(y) to order $\alpha$ or $\beta_{i}$ (i = 1, 2, 3) becomes the conventional n-th derivative of the function f(y). Therefore, specializing the result of Podlubny^8^ to n = 1, for $\alpha$ and $\beta_{i}$ → 1, (i = 1, 2, 3), the continuity equation of fluid flow transforms into

17 $$\frac{{\partial \rho \left( {\overline{x}, t} \right)}}{\partial t} = - \mathop \sum \limits_{i = 1}^{3} \frac{\partial }{{\partial x_{i} }}\left( {\rho \left( {\overline{x}, t} \right)u_{i} \left( {\overline{x}, t} \right)} \right),\overline{x} = \left( {x_{1} ,x_{2} , x_{3} } \right),$$

which is the conventional continuity equation for fluid flow in integer time-space^50,51^.

From the general fractional continuity Eqs. (14) or (15) for fluid flow it follows that the continuity equation for incompressible fluid flow with constant density in fractional time and multi-fractional space reduces to the form

18 $$\mathop \sum \limits_{i = 1}^{3} \frac{{\Gamma \left( {2 - \beta_{i} } \right)}}{{x_{i}^{{1 - \beta_{i} }} }} \left( {\frac{\partial }{{\partial x_{i} }}} \right)^{{\beta_{i} }} \left( {u_{i} \left( {\overline{x}, t} \right)} \right) = 0$$

which when $\alpha$ and $\beta_{i}$ → 1 (i = 1, 2, 3) results in

19 $$\mathop \sum \limits_{i = 1}^{3} \frac{\partial }{{\partial x_{i} }}\left( {u_{i} \left( {\overline{x}, t} \right)} \right) = 0$$

which is the conventional continuity equation for incompressible fluid flow.

### Momentum equations of unsteady flow in fractional time and multifractional space

The net momentum flux through the control volume in Fig. 2 may be expressed as:

20 $$\begin{aligned} & \left[ {\rho \left. {u_{i} u_{1} } \right|_{{\left( {x_{1} ~,x_{2} ,~x_{3} ;t} \right)}} - \left. {\rho u_{i} u_{1} } \right|_{{\left( {x_{1} - \Delta x_{1} ~,x_{2} ,~x_{3} ;t} \right)}} } \right]\Delta x_{2} \Delta x_{3} + \left[ {\left. {\rho u_{i} u_{2} } \right|_{{\left( {x_{1} ,x_{2} ,~x_{3} ;t} \right)}} - \left. {\rho u_{i} u_{2} } \right|_{{\left( {x_{1} ,x_{2} - \Delta x_{2} ,~x_{3} ;t} \right)}} } \right]\Delta x_{1} \Delta x_{3} \\ & \;\; + \left[ {\left. {\rho u_{i} u_{3} } \right|_{{\left( {x_{1} ~,x_{2} ,~x_{3} ;t} \right)}} - \left. {\rho u_{i} u_{3} } \right|_{{\left( {x_{1} ~,x_{2} ,~x_{3} - \Delta x_{3} ;t} \right)}} } \right]\Delta x_{1} \Delta x_{2} . \\ \end{aligned}$$

along directions *x*_*i*_, i = 1,2,3. Meanwhile the net pressure forces on the control volume surface may be expressed as:

21 $$\begin{gathered} \left( {P|_{{\left( {x_{1} ,x_{2} , x_{3} ;t} \right)}} - P|_{{\left( {x_{1} - \Delta x_{1} ,x_{2} , x_{3} ;t} \right)}} } \right)\Delta x_{2} \Delta x_{3} \hfill \\ \left( {P|_{{\left( {x_{1} ,x_{2} , x_{3} ;t} \right)}} - P|_{{\left( {x_{1} ,x_{2} - \Delta x_{2} , x_{3} ;t} \right)}} } \right)\Delta x_{1} \Delta x_{3} \hfill \\ \left( {P|_{{\left( {x_{1} ,x_{2} , x_{3} ;t} \right)}} - P|_{{\left( {x_{1} ,x_{2} , x_{3} - \Delta x_{3} ;t} \right)}} } \right) \Delta x_{1} \Delta x_{2} \hfill \\ \end{gathered}$$

**Figure 3:**
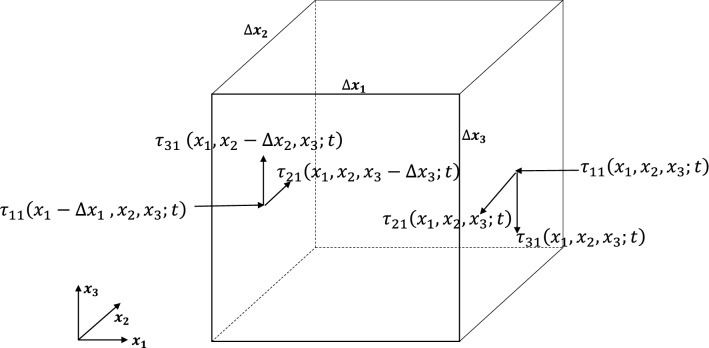
Surface forces in the momentum equation.

Again, referring to Fig. 3, the net stress (viscous) forces on the control volume surface may be expressed as:

22 $$\begin{aligned} & \left( {\tau_{i1} |_{{\left( {x_{1} ,x_{2} , x_{3} ;t} \right)}} - \tau_{i1} |_{{\left( {x_{1} - \Delta x_{1} ,x_{2} , x_{3} ;t} \right)}} } \right)\Delta x_{2} \Delta x_{3} + \left( {\tau_{i2} |_{{\left( {x_{1} ,x_{2} , x_{3} ;t} \right)}} - \tau_{i2} |_{{\left( {x_{1} ,x_{2} - \Delta x_{2} , x_{3} ;t} \right)}} } \right)\Delta x_{1} \Delta x_{3} \\ & \;\; + \left( {\tau_{i3} |_{{\left( {x_{1} ,x_{2} , x_{3} ;t} \right)}} - \tau_{i3} |_{{\left( {x_{1} ,x_{2} , x_{3} - \Delta x_{3} ;t} \right)}} } \right) \Delta x_{1} \Delta x_{2} \\ \end{aligned}$$

along directions *x*_*i*_, i = 1,2,3. Finally, the body force on the control volume may be expressed as:

23 $$- \rho F_{i} \Delta x_{1} \Delta x_{2} \Delta x_{3} ,\;{\text{i}} = {1},{2},{3}.$$

By introducing Eq. (4) into Eqs. (20)–(23) with $\Delta x_{i}$ = $x_{i} ,$ i = 1,2,3, and expressing the resulting Caputo derivative $_0D_{x}^{\beta } f\left( x \right){ }$ by $\frac{{\partial^{\beta } f\left( x \right)}}{{\left( {\partial x} \right)^{\beta } }}$, the net momentum flux in the $x_{i}$ direction, may be expressed to $\beta_{i}$-order, i = 1,2,3, as:

24 $$\begin{aligned} \frac{{\left( {\Delta x_{1} } \right)^{{\beta_{1} }} }}{{\Gamma \left( {\beta_{1} + 1} \right)}} & \left( {\frac{\partial }{{\partial x_{1} }}} \right)^{{\beta_{1} }} \left( {\rho \left( {\overline{x};t} \right)u_{i} \left( {\overline{x};t} \right)u_{1} \left( {\overline{x};t} \right)} \right)\Delta x_{2} \Delta x_{3} \\ & \;\; + \frac{{\left( {\Delta x_{2} } \right)^{{\beta_{2} }} }}{{\Gamma \left( {\beta_{2} + 1} \right)}}\left( {\frac{\partial }{{\partial x_{2} }}} \right)^{{\beta_{2} }} \left( {\rho \left( {\overline{x};t} \right)u_{i} \left( {\overline{x};t} \right)u_{2} \left( {\overline{x};t} \right)} \right)\Delta x_{1} \Delta x_{3} \\ & \;\; + \frac{{\left( {\Delta x_{3} } \right)^{{\beta_{3} }} }}{{\Gamma \left( {\beta_{3} + 1} \right)}}\left( {\frac{\partial }{{\partial x_{3} }}} \right)^{{\beta_{3} }} \left( {\rho \left( {\overline{x};t} \right)u_{i} \left( {\overline{x};t} \right)u_{3} \left( {\overline{x};t} \right)} \right)\Delta x_{1} \Delta x_{2} \\ & \;\; + \frac{{\left( {\Delta x_{i} } \right)^{{\beta_{i} }} }}{{\Gamma \left( {\beta_{i} + 1} \right)}}\left( {\frac{\partial }{{\partial x_{i} }}} \right)^{{\beta_{i} }} \left( {P\left( {\overline{x};t} \right)} \right)\Delta x_{j} \Delta x_{k} \\ & \;\; + \frac{{\left( {\Delta x_{1} } \right)^{{\beta_{1} }} }}{{\Gamma \left( {\beta_{1} + 1} \right)}}\left( {\frac{\partial }{{\partial x_{1} }}} \right)^{{\beta_{1} }} \left( {\tau_{i1} \left( {\overline{x};t} \right)} \right)\Delta x_{2} \Delta x_{3} \\ & \;\; + \frac{{\left( {\Delta x_{2} } \right)^{{\beta_{2} }} }}{{\Gamma \left( {\beta_{2} + 1} \right)}}\left( {\frac{\partial }{{\partial x_{2} }}} \right)^{{\beta_{2} }} \left( {\tau_{i2} \left( {\overline{x};t} \right)} \right)\Delta x_{1} \Delta x_{3} \\ & \;\; + \frac{{\left( {\Delta x_{3} } \right)^{{\beta_{3} }} }}{{\Gamma \left( {\beta_{3} + 1} \right)}}\left( {\frac{\partial }{{\partial x_{3} }}} \right)^{{\beta_{3} }} \left( {\tau_{i3} \left( {\overline{x};t} \right)} \right)\Delta x_{1} \Delta x_{2} - \rho F_{i} \Delta x_{1} \Delta x_{2} \Delta x_{3} , \\ & \;\;{\text{i}} = {1},{2},{3},\,\,j \ne k \ne i,\,\, j + k + i = 3, \,\,1 \le j,\,\,k \le 3,\;j{ }\;and\;{ }k{ }\ integers. \\ \end{aligned}$$

Introducing Eq. (7) into Eq. (24) results in the net momentum flux expressed with respect to $\beta_{i}$-order fractional space in the *x*_*i*_ direction, i = 1,2,3, as:

25 $$\begin{aligned} \frac{{\Gamma \left( {2 - \beta_{1} } \right)}}{{x_{1}^{{1 - \beta_{1} }} }} & \left( {\frac{\partial }{{\partial x_{1} }}} \right)^{{\beta_{1} }} \left( {\rho \left( {\overline{x};t} \right)u_{i} \left( {\overline{x};t} \right)u_{1} \left( {\overline{x};t} \right)} \right)\Delta x_{1} \Delta x_{2} \Delta x_{3} \\ & \;\; + \frac{{\Gamma \left( {2 - \beta_{2} } \right)}}{{x_{2}^{{1 - \beta_{2} }} }}\left( {\frac{\partial }{{\partial x_{2} }}} \right)^{{\beta_{2} }} \left( {\rho \left( {\overline{x};t} \right)u_{i} \left( {\overline{x};t} \right)u_{2} \left( {\overline{x};t} \right)} \right)\Delta x_{1} \Delta x_{2} \Delta x_{3} \\ & \;\; + \frac{{\Gamma \left( {2 - \beta_{3} } \right)}}{{x_{3}^{{1 - \beta_{3} }} }}\left( {\frac{\partial }{{\partial x_{3} }}} \right)^{{\beta_{3} }} \left( {\rho \left( {\overline{x};t} \right)u_{i} \left( {\overline{x};t} \right)u_{3} \left( {\overline{x};t} \right)} \right)\Delta x_{1} \Delta x_{2} \Delta x_{3} \\ & \;\; + \frac{{\Gamma \left( {2 - \beta_{i} } \right)}}{{x_{i}^{{1 - \beta_{i} }} }}\left( {\frac{\partial }{{\partial x_{i} }}} \right)^{{\beta_{i} }} \left( {P\left( {\overline{x};t} \right)} \right)\Delta x_{1} \Delta x_{2} \Delta x_{3} \\ & \;\; + \frac{{\Gamma \left( {2 - \beta_{1} } \right)}}{{x_{1}^{{1 - \beta_{1} }} }}\left( {\frac{\partial }{{\partial x_{1} }}} \right)^{{\beta_{1} }} \left( {\tau_{i1} \left( {\overline{x};t} \right)} \right)\Delta x_{1} \Delta x_{2} \Delta x_{3} \\ & \;\; + \frac{{\Gamma \left( {2 - \beta_{2} } \right)}}{{x_{2}^{{1 - \beta_{2} }} }}\left( {\frac{\partial }{{\partial x_{2} }}} \right)^{{\beta_{2} }} \left( {\tau_{i2} \left( {\overline{x};t} \right)} \right)\Delta x_{1} \Delta x_{2} \Delta x_{3} \\ & \;\; + \frac{{\Gamma \left( {2 - \beta_{3} } \right)}}{{x_{3}^{{1 - \beta_{3} }} }}\left( {\frac{\partial }{{\partial x_{3} }}} \right)^{{\beta_{3} }} \left( {\tau_{i3} \left( {\overline{x};t} \right)} \right)\Delta x_{1} \Delta x_{2} \Delta x_{3} \\ & \;\; - \rho \left( {\overline{x};t} \right)F_{i} \left( {\overline{x};t} \right)\Delta x_{1} \Delta x_{2} \Delta x_{3} ,\;{\text{i}} = {1},{2},{3}. \\ \end{aligned}$$

Meanwhile, the change of momentum within the control volume during (t−Δt, t) may be expressed as:

26 $$\left[ {\rho \left( {\overline{x}, t} \right)u_{i} \left( {\overline{x};t} \right) - \rho \left( {\overline{x}, t - \Delta t} \right)u_{i} \left( {\overline{x};t - \Delta t} \right)} \right] \Delta x_{1} \Delta x_{2} \Delta x_{3} .$$

Hence, the time rate of change of momentum within the control volume may be expressed as:

27 $$\frac{{\left[ {\rho \left( {\overline{x}, t} \right)u_{i} \left( {\overline{x};t} \right) - \rho \left( {\overline{x}, t - \Delta t} \right)u_{i} \left( {\overline{x};t - \Delta t} \right)} \right]}}{\Delta t} \Delta x_{1} \Delta x_{2} \Delta x_{3} ,\;\,\,{\text{i}} = {1},{2},{3}.$$

Following the same procedure that led to Eqs. (10)–(12) with $\rho u_{i}$ replacing $\rho$ in these equations, one obtains the time rate of change of momentum within the control volume with respect to $\alpha$-order fractional time increments as:

28 $$\frac{{\Gamma \left( {2 - \alpha } \right)}}{{t^{1 - \alpha } }} \frac{{\partial^{\alpha } \rho \left( {\overline{x}, t} \right)u_{i} \left( {\overline{x};t} \right)}}{{\left( {\partial t} \right)^{\alpha } }}\Delta x_{1} \Delta x_{2} \Delta x_{3},\,\,{\text{i}} = {1},{2},{3}.$$

Since the time rate of momentum change within the control volume is inversely related to the net momentum flux through the control volume, Eqs. (25) and (28) can be combined to yield

29 $$\begin{aligned} & \frac{{\Gamma \left( {2 - \alpha } \right)}}{{t^{1 - \alpha } }} \frac{{\partial^{\alpha } \rho \left( {\overline{x}, t} \right)u_{i} \left( {\overline{x};t} \right)}}{{\left( {\partial t} \right)^{\alpha } }} = - \mathop \sum \limits_{j = 1}^{3} \frac{{\Gamma \left( {2 - \beta_{j} } \right)}}{{x_{j}^{{1 - \beta_{j} }} }}\left( {\frac{\partial }{{\partial x_{j} }}} \right)^{{\beta_{j} }} \left( {\rho \left( {\overline{x}, t} \right)u_{i} \left( {\overline{x};t} \right)u_{j} \left( {\overline{x};t} \right)} \right) \\ & \;\;\; - \frac{{\Gamma \left( {2 - \beta_{i} } \right)}}{{x_{i}^{{1 - \beta_{i} }} }}\left( {\frac{\partial }{{\partial x_{i} }}} \right)^{{\beta_{i} }} \left( {P\left( {\overline{x}, t} \right)} \right) + \mathop \sum \limits_{j = 1}^{3} \frac{{\Gamma \left( {2 - \beta_{j} } \right)}}{{x_{j}^{{1 - \beta_{j} }} }}\left( {\frac{\partial }{{\partial x_{j} }}} \right)^{{\beta_{j} }} \left( {\tau_{ij} \left( {\overline{x};t} \right)} \right) + \rho F_{i} \left( {\overline{x}, t} \right), \\ & \;\;\;{\text{i}} = {1},{2},{3} \\ \end{aligned}$$

as the general momentum equation of unsteady fluid flow in fractional time and multi-fractional space. The body force *F*_*i*_, i = 1,2,3, can be interpreted as the gravitational force *g*_*i*_, i = 1,2,3^50^. Under this interpretation the general momentum equations of unsteady fluid flow in fractional time and multi-fractional space may also be expressed as

30 $$\begin{aligned} & \frac{{\Gamma \left( {2 - \alpha } \right)}}{{t^{1 - \alpha } }} \frac{{\partial^{\alpha } \rho \left( {\overline{x}, t} \right)u_{i} \left( {\overline{x};t} \right)}}{{\left( {\partial t} \right)^{\alpha } }} = - \mathop \sum \limits_{j = 1}^{3} \frac{{\Gamma \left( {2 - \beta_{j} } \right)}}{{x_{j}^{{1 - \beta_{j} }} }}\left( {\frac{\partial }{{\partial x_{j} }}} \right)^{{\beta_{j} }} \left( {\rho \left( {\overline{x}, t} \right)u_{i} \left( {\overline{x};t} \right)u_{j} \left( {\overline{x};t} \right)} \right) \\ & \;\; - \frac{{\Gamma \left( {2 - \beta_{i} } \right)}}{{x_{i}^{{1 - \beta_{i} }} }}\left( {\frac{\partial }{{\partial x_{i} }}} \right)^{{\beta_{i} }} \left( {P\left( {\overline{x}, t} \right)} \right) + \mathop \sum \limits_{j = 1}^{3} \frac{{\Gamma \left( {2 - \beta_{j} } \right)}}{{x_{j}^{{1 - \beta_{j} }} }}\left( {\frac{\partial }{{\partial x_{j} }}} \right)^{{\beta_{j} }} \left( {\tau_{ij} \left( {\overline{x};t} \right)} \right) + \rho g_{i} \left( {\overline{x}, t} \right), \\ & \;\;{\text{i}} = {1},{2},{3} \\ \end{aligned}$$

where the surface forces $\tau_{ij}$ will need to be defined in terms of flow velocities differently for incompressible and compressible fluid flows for the closure of the equation. This issue will be addressed later in the study.

The fractional momentum Eq. (30) for incompressible fluid flow can also be written as

31 $$\begin{aligned} & \frac{{\partial^{\alpha } \rho \left( {\overline{x}, t} \right)u_{i} \left( {\overline{x};t} \right)}}{{\left( {\partial t} \right)^{\alpha } }} = - \mathop \sum \limits_{j = 1}^{3} \frac{{\Gamma \left( {2 - \beta_{j} } \right)}}{{x_{j}^{{1 - \beta_{j} }} }}\frac{{t^{1 - \alpha } }}{{\Gamma \left( {2 - \alpha } \right)}}\left( {\frac{\partial }{{\partial x_{j} }}} \right)^{{\beta_{j} }} \left( {\rho \left( {\overline{x}, t} \right)u_{i} \left( {\overline{x};t} \right)u_{j} \left( {\overline{x};t} \right)} \right) \\ & \;\; - \frac{{\Gamma \left( {2 - \beta_{i} } \right)}}{{x_{i}^{{1 - \beta_{i} }} }}\frac{{t^{1 - \alpha } }}{{\Gamma \left( {2 - \alpha } \right)}}\left( {\frac{\partial }{{\partial x_{i} }}} \right)^{{\beta_{i} }} \left( {P\left( {\overline{x}, t} \right)} \right) + \mathop \sum \limits_{j = 1}^{3} \frac{{\Gamma \left( {2 - \beta_{j} } \right)}}{{x_{j}^{{1 - \beta_{j} }} }}\frac{{t^{1 - \alpha } }}{{\Gamma \left( {2 - \alpha } \right)}}\left( {\frac{\partial }{{\partial x_{j} }}} \right)^{{\beta_{j} }} \left( {\tau_{ij} \left( {\overline{x};t} \right)} \right) \\ & \;\; + \frac{{t^{1 - \alpha } }}{{\Gamma \left( {2 - \alpha } \right)}}\rho g_{i} \left( {\overline{x}, t} \right) \\ \end{aligned}$$

### Euler’s equation in fractional time–space

If the fluid flow has no friction, that is, if the shearing stresses $\tau_{ij}$ are zero and the normal forces are simply the pressure forces, the flow system is called inviscid. Then Eqs. (30) or (31) simplify to

32 $$\begin{aligned} \frac{{\partial^{\alpha } \rho \left( {\overline{x}, t} \right)u_{i} \left( {\overline{x};t} \right)}}{{\left( {\partial t} \right)^{\alpha } }} & = - \mathop \sum \limits_{j = 1}^{3} \frac{{\Gamma \left( {2 - \beta_{j} } \right)}}{{x_{j}^{{1 - \beta_{j} }} }}\frac{{t^{1 - \alpha } }}{{\Gamma \left( {2 - \alpha } \right)}}\left( {\frac{\partial }{{\partial x_{j} }}} \right)^{{\beta_{j} }} \left( {\rho \left( {\overline{x}, t} \right)u_{i} \left( {\overline{x};t} \right)u_{j} \left( {\overline{x};t} \right)} \right) \\ & \;\; - \frac{{\Gamma \left( {2 - \beta_{i} } \right)}}{{x_{i}^{{1 - \beta_{i} }} }}\frac{{t^{1 - \alpha } }}{{\Gamma \left( {2 - \alpha } \right)}}\left( {\frac{\partial }{{\partial x_{i} }}} \right)^{{\beta_{i} }} \left( {P\left( {\overline{x}, t} \right)} \right) + \frac{{t^{1 - \alpha } }}{{\Gamma \left( {2 - \alpha } \right)}}\rho g_{i} \left( {\overline{x}, t} \right),{\text{i}} = {1},{2},{3}. \\ \end{aligned}$$

In analogy to Euler’s equation for inviscid fluid flow in integer time–space, Eq. (32) may be called as “Euler’s equation for fluid flow in fractional time–space”.

### Momentum equations for incompressible fluid flow under Stokes viscosity law in fractional time and multifractional space

For incompressible fluid flow, the shear stresses can be expressed in terms of flow velocities using Stokes viscosity law as^50^:

33 $$\tau_{ii} = 2\mu \frac{{\partial u_{i} }}{{\partial x_{i} }},\;\;{\text{i}} = {1},{2},{3}$$

34 $$\tau_{ij} = \tau_{ji} = \mu \left( {\frac{{\partial u_{i} }}{{\partial x_{j} }} + \frac{{\partial u_{j} }}{{\partial x_{i} }}} \right),\,\;{\text{i}},{\text{j}} = {1},{2},{3}$$

in the conventional integer space. In Eqs. (33) and (34) $\mu$ is the viscosity coefficient. Then using Eqs. (4) and (7) on Eqs. (33) and (34) results in

35 $$\tau_{ii} = 2\mu \left( {\frac{{{\Gamma }\left( {2 - \beta_{i} } \right)}}{{x_{i}^{{1 - \beta_{i} }} }}\frac{{\partial^{{\beta_{i} }} u_{i} }}{{\left( {\partial x_{i} } \right)^{{\beta_{i} }} }}} \right) = \mu \left[ {\frac{{{\Gamma }\left( {2 - \beta_{i} } \right)}}{{x_{i}^{{1 - \beta_{i} }} }}\frac{{\partial^{{\beta_{i} }} u_{i} }}{{\left( {\partial x_{i} } \right)^{{\beta_{i} }} }} + \frac{{{\Gamma }\left( {2 - \beta_{i} } \right)}}{{x_{i}^{{1 - \beta_{i} }} }}\frac{{\partial^{{\beta_{i} }} u_{i} }}{{\left( {\partial x_{i} } \right)^{{\beta_{i} }} }}} \right],\;\;{\text{i}} = {1},{2},{3}$$

and

36 $$\tau_{ij} = \tau_{ji} = \mu \left[ {\frac{{{\Gamma }\left( {2 - \beta_{j} } \right)}}{{x_{j}^{{1 - \beta_{j} }} }}\frac{{\partial^{{\beta_{j} }} u_{i} }}{{\left( {\partial x_{j} } \right)^{{\beta_{j} }} }} + \frac{{{\Gamma }\left( {2 - \beta_{i} } \right)}}{{x_{i}^{{1 - \beta_{i} }} }}\frac{{\partial^{{\beta_{i} }} u_{j} }}{{\left( {\partial x_{i} } \right)^{{\beta_{i} }} }}} \right],\;\;{\text{i}},{\text{j}} = {1},{2},{3}$$

for the Stokes viscosity relations in multi-fractional space.

Substituting Eqs. (35) and (36) into Eq. (29) and noting the fluid density is constant for incompressible fluid flow results in the momentum equations

37 $$\begin{aligned} \rho \frac{{\Gamma \left( {2 - \alpha } \right)}}{{t^{1 - \alpha } }} \frac{{\partial^{\alpha } u_{i} \left( {\overline{x};t} \right)}}{{\left( {\partial t} \right)^{\alpha } }} & = - \rho \mathop \sum \limits_{j = 1}^{3} \frac{{\Gamma \left( {2 - \beta_{j} } \right)}}{{x_{j}^{{1 - \beta_{j} }} }}\left( {\frac{\partial }{{\partial x_{j} }}} \right)^{{\beta_{j} }} \left( {u_{i} \left( {\overline{x};t} \right)u_{j} \left( {\overline{x};t} \right)} \right) \\ & \;\; + \rho g_{i} \left( {\overline{x}, t} \right) - \frac{{\Gamma \left( {2 - \beta_{i} } \right)}}{{x_{i}^{{1 - \beta_{i} }} }}\left( {\frac{\partial }{{\partial x_{i} }}} \right)^{{\beta_{i} }} \left( {P\left( {\overline{x}, t} \right)} \right) \\ & \;\; + \mu \mathop \sum \limits_{j = 1}^{3} \frac{{\Gamma \left( {2 - \beta_{j} } \right)}}{{x_{j}^{{1 - \beta_{j} }} }}\left( {\frac{\partial }{{\partial x_{j} }}} \right)^{{\beta_{j} }} \left( {\frac{{{\Gamma }\left( {2 - \beta_{j} } \right)}}{{x_{j}^{{1 - \beta_{j} }} }}\frac{{\partial^{{\beta_{j} }} u_{i} }}{{\left( {\partial x_{j} } \right)^{{\beta_{j} }} }} + \frac{{{\Gamma }\left( {2 - \beta_{i} } \right)}}{{x_{i}^{{1 - \beta_{i} }} }}\frac{{\partial^{{\beta_{i} }} u_{j} }}{{\left( {\partial x_{i} } \right)^{{\beta_{i} }} }}} \right),i = {1},{2},{3}. \\ \end{aligned}$$

Next, the flow velocities $u_{i} \left( {\overline{x};t} \right)$ (*i* = 1,2,3) are considered analytic functions, and it is noted that the fractional scaling powers in time and space are between zero and one, that is 0 $< \alpha ,\beta_{i} < 1$, (i = 1,2,3). As such, the Caputo fractional derivatives $\left( {\frac{\partial }{{\partial x_{j} }}} \right)^{{\beta_{j} }}$ and $\left( {\frac{\partial }{{\partial x_{i} }}} \right)^{{\beta_{i} }}$ that operate on the flow velocities $u_{j} \left( {\overline{x};t} \right)$ (*j* = 1,2,3) will commute^52^. Combining this property of the above Caputo fractional derivatives on the last line on the right-hand-side of Eq. (37) with the fractional continuity Eq. (18) for incompressible fluid flow results in the governing equation

38 $$\begin{aligned} \rho \frac{{\Gamma \left( {2 - \alpha } \right)}}{{t^{1 - \alpha } }} \frac{{\partial^{\alpha } u_{i} \left( {\overline{x};t} \right)}}{{\left( {\partial t} \right)^{\alpha } }} & = - \rho \mathop \sum \limits_{j = 1}^{3} \frac{{\Gamma \left( {2 - \beta_{j} } \right)}}{{x_{j}^{{1 - \beta_{j} }} }}\left( {\frac{\partial }{{\partial x_{j} }}} \right)^{{\beta_{j} }} \left( {u_{i} \left( {\overline{x};t} \right)u_{j} \left( {\overline{x};t} \right)} \right) \\ & \;\; + \rho g_{i} \left( {\overline{x}, t} \right) - \frac{{\Gamma \left( {2 - \beta_{i} } \right)}}{{x_{i}^{{1 - \beta_{i} }} }}\left( {\frac{\partial }{{\partial x_{i} }}} \right)^{{\beta_{i} }} \left( {P\left( {\overline{x}, t} \right)} \right) \\ & \;\; + \mu \mathop \sum \limits_{j = 1}^{3} \frac{{\Gamma \left( {2 - \beta_{j} } \right)}}{{x_{j}^{{1 - \beta_{j} }} }}\left( {\frac{\partial }{{\partial x_{j} }}} \right)^{{\beta_{j} }} \left( {\frac{{{\Gamma }\left( {2 - \beta_{j} } \right)}}{{x_{j}^{{1 - \beta_{j} }} }}\frac{{\partial^{{\beta_{j} }} u_{i} }}{{\left( {\partial x_{j} } \right)^{{\beta_{j} }} }}} \right),{\text{i}} = {1},{2},{3} \\ \end{aligned}$$

as the momentum equations for incompressible fluid flow with constant density $\rho$ and constant viscosity $\mu$ in $\beta_{i}$-scaled (i = 1,2,3) multi-fractional space and in α-scaled fractional time.

As mentioned earlier, Podlubny^8^ has shown that for $n$−1 < $\alpha ,\beta_{i} < n$ where n is any positive integer, as $\alpha$ and $\beta_{i}$ $\to$ n, the Caputo fractional derivative of a function f(y) to order $\alpha$ or $\beta_{i}$ (i = 1, 2, 3) becomes the conventional n-th derivative of the function f(y). Therefore, specializing the result of Podlubny^8^ to n = 1, for $\alpha$ or $\beta_{i}$ → 1, (i = 1, 2, 3), the fractional momentum Eqs. (38) of incompressible fluid flow transform into

39 $$\rho \frac{{\partial u_{i} \left( {\overline{x};t} \right)}}{\partial t} = - \rho \mathop \sum \limits_{j = 1}^{3} \frac{\partial }{{\partial x_{j} }}\left( {u_{i} \left( {\overline{x};t} \right)u_{j} \left( {\overline{x};t} \right)} \right) + \rho g_{i} \left( {\overline{x}, t} \right) - \frac{\partial }{{\partial x_{i} }}\left( {P\left( {\overline{x}, t} \right)} \right) + \mu \mathop \sum \limits_{j = 1}^{3} \frac{{\partial^{2} u_{i} }}{{\partial x_{j}^{2} }},\;\;{\text{i}} = {1},{2},{3}$$

which reduce to

40 $$\rho \frac{{\partial u_{i} \left( {\overline{x};t} \right)}}{\partial t} = - \rho \mathop \sum \limits_{j = 1}^{3} u_{j} \left( {\overline{x};t} \right)\frac{\partial }{{\partial x_{j} }}\left( {u_{i} \left( {\overline{x};t} \right)} \right) + \rho g_{i} \left( {\overline{x}, t} \right) - \frac{\partial }{{\partial x_{i} }}\left( {P\left( {\overline{x}, t} \right)} \right) + \mu \mathop \sum \limits_{j = 1}^{3} \frac{{\partial^{2} u_{i} }}{{\partial x_{j}^{2} }},\;\;{\text{i}} = {1},{2},{3}$$

which are the conventional Navier–Stokes equations for Newtonian, incompressible fluid flow with constant density and viscosity^50^. Within this framework, the Eq. (38) for incompressible fluid flow in fractional time and multi-fractional space may be interpreted as the extension of the corresponding Navier–Stokes equations to fractional time and multi-fractional space.

### Momentum equations for compressible fluid flow under Stokes viscosity law in fractional time and multifractional space

In the case of compressible viscous fluid flow, while the shear stresses are the same as in incompressible flow, the Stokes relations need to be modified for the effect of volume change in the fluid due to compression, leading to the normal forces $\sigma_{ii}$ (*i* = 1,2,3) being expressed as^51,53^,

41 $$\sigma_{ii} = -p + \tau_{ii} - \frac{2}{3}\mu \mathop \sum \limits_{k = 1}^{3} \frac{{\partial u_{k} }}{{\partial x_{k} }}, \;\;i = {1},{2},{3}$$

42 $$\sigma_{ii} = -p + 2\mu \frac{{\partial u_{i} }}{{\partial x_{i} }} - \frac{2}{3}\mu \mathop \sum \limits_{k = 1}^{3} \frac{{\partial u_{k} }}{{\partial x_{k} }},\;\;i = {1},{2},{3}$$

Then using Eqs. (4) and (7) on Eq. (42) results in

43 $$\sigma_{ii} = -p + \mu \left[ {\frac{{{\Gamma }\left( {2 - \beta_{i} } \right)}}{{x_{i}^{{1 - \beta_{i} }} }}\frac{{\partial^{{\beta_{i} }} u_{i} }}{{\left( {\partial x_{i} } \right)^{{\beta_{i} }} }} + \frac{{{\Gamma }\left( {2 - \beta_{i} } \right)}}{{x_{i}^{{1 - \beta_{i} }} }}\frac{{\partial^{{\beta_{i} }} u_{i} }}{{\left( {\partial x_{i} } \right)^{{\beta_{i} }} }}} \right] - \frac{2}{3}\mu \mathop \sum \limits_{k = 1}^{3} \frac{{{\Gamma }\left( {2 - \beta_{k} } \right)}}{{x_{k}^{{1 - \beta_{k} }} }}\frac{{\partial^{{\beta_{k} }} u_{k} }}{{\left( {\partial x_{k} } \right)^{{\beta_{k} }} }},\;\;i = {1},{2},{3}$$

Combining Eqs. (36) and (43) with modified normal forces due to fluid compression, with Eq. (29) results in the fractional momentum equations

44 $$\begin{aligned} \frac{{\Gamma \left( {2 - \alpha } \right)}}{{t^{1 - \alpha } }} \frac{{\partial^{\alpha } \rho \left( {\overline{x}, t} \right)u_{i} \left( {\overline{x};t} \right)}}{{\left( {\partial t} \right)^{\alpha } }} & = - \mathop \sum \limits_{j = 1}^{3} \frac{{\Gamma \left( {2 - \beta_{j} } \right)}}{{x_{j}^{{1 - \beta_{j} }} }}\left( {\frac{\partial }{{\partial x_{j} }}} \right)^{{\beta_{j} }} \left( {\rho \left( {\overline{x}, t} \right)u_{i} \left( {\overline{x};t} \right)u_{j} \left( {\overline{x};t} \right)} \right) \\ & \;\; + \rho \left( {\overline{x}, t} \right)g_{i} \left( {\overline{x}, t} \right) - \frac{{\Gamma \left( {2 - \beta_{i} } \right)}}{{x_{i}^{{1 - \beta_{i} }} }}\left( {\frac{\partial }{{\partial x_{i} }}} \right)^{{\beta_{i} }} \left( {P\left( {\overline{x}, t} \right)} \right) \\ & \;\; + \mathop \sum \limits_{j = 1}^{3} \frac{{\Gamma \left( {2 - \beta_{j} } \right)}}{{x_{j}^{{1 - \beta_{j} }} }}\left( {\frac{\partial }{{\partial x_{j} }}} \right)^{{\beta_{j} }} \left[ {\mu \frac{{{\Gamma }\left( {2 - \beta_{j} } \right)}}{{x_{j}^{{1 - \beta_{j} }} }}\frac{{\partial^{{\beta_{j} }} u_{i} }}{{\left( {\partial x_{j} } \right)^{{\beta_{j} }} }} + \mu \frac{{{\Gamma }\left( {2 - \beta_{i} } \right)}}{{x_{i}^{{1 - \beta_{i} }} }}\frac{{\partial^{{\beta_{i} }} u_{j} }}{{\left( {\partial x_{i} } \right)^{{\beta_{i} }} }}} \right] \\ & \;\; - \frac{{\Gamma \left( {2 - \beta_{i} } \right)}}{{x_{i}^{{1 - \beta_{i} }} }}\left( {\frac{\partial }{{\partial x_{i} }}} \right)^{{\beta_{i} }} \left[ {\frac{2}{3}\mu \mathop \sum \limits_{k = 1}^{3} \frac{{{\Gamma }\left( {2 - \beta_{k} } \right)}}{{x_{k}^{{1 - \beta_{k} }} }}\frac{{\partial^{{\beta_{k} }} u_{k} }}{{\left( {\partial x_{k} } \right)^{{\beta_{k} }} }}} \right], i = {1},{2},{3} \\ \end{aligned}$$

for compressible fluid flow in $\beta_{i}$-scaled (i = 1,2,3) multi-fractional space and in α-scaled fractional time.

Again specializing the result of Podlubny^8^ to n = 1, for $\alpha$ or $\beta_{i}$ → 1, (i = 1, 2, 3), that the Caputo fractional derivative of a function f(y) to order $\alpha$ or $\beta_{i}$ (i = 1, 2, 3) becomes the conventional derivative of the function f(y), and applying it to Eq. (44) results in

45 $$\begin{aligned} \frac{{\partial \rho \left( {\overline{x}, t} \right)u_{i} \left( {\overline{x};t} \right)}}{\partial t} & = - \mathop \sum \limits_{j = 1}^{3} \frac{\partial }{{\partial x_{j} }}\left( {\rho \left( {\overline{x}, t} \right)u_{i} \left( {\overline{x};t} \right)u_{j} \left( {\overline{x};t} \right)} \right) \\ & \;\; + \rho \left( {\overline{x}, t} \right)g_{i} \left( {\overline{x}, t} \right) - \frac{\partial }{{\partial x_{i} }}\left( {P\left( {\overline{x}, t} \right)} \right) \\ & \;\; + \mathop \sum \limits_{j = 1}^{3} \frac{\partial }{{\partial x_{j} }}\left[ {\mu \frac{{\partial u_{i} }}{{\partial x_{j} }} + \mu \frac{{\partial u_{j} }}{{\partial x_{i} }}} \right] - \frac{\partial }{{\partial x_{i} }} \left[ {\frac{2}{3}\mu \mathop \sum \limits_{k = 1}^{3} \frac{{\partial u_{k} }}{{\partial x_{k} }}} \right], i = {1},{2},{3} \\ \end{aligned}$$

which are consistent with the conventional Navier–Stokes momentum equations for compressible flow^54^. Introducing the continuity Eq. (17) into Eq. (45) results in

46 $$\begin{aligned} \rho \left( {\overline{x}, t} \right)\frac{{\partial u_{i} \left( {\overline{x};t} \right)}}{\partial t} & = - \rho \left( {\overline{x}, t} \right)\mathop \sum \limits_{j = 1}^{3} u_{j} \left( {\overline{x};t} \right)\frac{\partial }{{\partial x_{j} }}\left( {u_{i} \left( {\overline{x};t} \right)} \right) \\ & \;\; + \rho \left( {\overline{x}, t} \right)g_{i} \left( {\overline{x}, t} \right) - \frac{\partial }{{\partial x_{i} }}\left( {P\left( {\overline{x}, t} \right)} \right) \\ & \;\; + \mathop \sum \limits_{j = 1}^{3} \frac{\partial }{{\partial x_{j} }}\left[ {\mu \frac{{\partial u_{i} }}{{\partial x_{j} }} + \mu \frac{{\partial u_{j} }}{{\partial x_{i} }}} \right] - \frac{\partial }{{\partial x_{i} }} \left[ {\frac{2}{3}\mu \mathop \sum \limits_{k = 1}^{3} \frac{{\partial u_{k} }}{{\partial x_{k} }}} \right], i = {1},{2},{3} \\ \end{aligned}$$

which are the conventional Navier–Stokes equations for compressible flow in detailed form. Hence, the conventional Navier–Stokes equations system (46) for compressible flow can be interpreted as the special case of the fractional equations system (44) for compressible flow in fractional time and multi-fractional space when the fractional powers become unity.

### Numerical application

In order to investigate the capabilities of the proposed fractional governing equations of hydrodynamics, the first Stokes Problem (i.e., flow due to a wall suddenly set into motion) is selected. A fluid with constant density and viscosity is bounded by a solid wall (at $x_{2} = 0$), which is set in motion in positive x_1_ direction at t = 0 with a constant velocity U_0_. There is no pressure gradient or gravity force in x_1_ direction. Then the conventional Navier–Stokes Equation (NSE) for this situation may be expressed as (see example 4.1–1 in Bird et al.^55^):

47 $$\rho \frac{{\partial u_{1} \left( {x_{2} ,t} \right)}}{\partial t} = \mu \frac{{\partial^{2} u_{1} \left( {x_{2} ,t} \right)}}{{\partial x_{2}^{2} }}$$

where the initial and boundary conditions are

48 $$\begin{gathered} u_{1} \left( {x_{2} ,t = 0} \right) = 0; \hfill \\ u_{1} \left( {x_{2} = 0,t \ge 0} \right) = U_{0} ; \hfill \\ u_{1} \left( {x_{2} = \infty ,t \ge 0} \right) = 0. \hfill \\ \end{gathered}$$

This is a simple unsteady flow problem with analytical solution (Equation 4.1-15 in Bird et al.^55^)

49 $$u_{1} \left( {x_{2} ,t} \right) = U_{0} \cdot erfc\left( {\frac{{x_{2} }}{{\sqrt {4\nu t} }}} \right)$$

where $erfc$ is the complementary error function (i.e., ${\text{erfc}}\left( {\text{x}} \right) = 1 - {\text{erf}}\left( {\text{x}} \right)$) and $\nu = \mu /\rho$. In this application, viscosity $\nu$ is 0.001 m^2^/s, total simulation time T is 5 h, and velocity $U_{0}$ is 1 m/s. The fractional form of this problem can be written as

50 $$\frac{{\Gamma \left( {2 - \alpha } \right)}}{{t^{1 - \alpha } }} \frac{{\partial^{\alpha } u_{1} \left( {x_{2} ,t} \right)}}{{\left( {\partial t} \right)^{\alpha } }} = { }\nu \frac{{\Gamma \left( {2 - \beta } \right)}}{{x_{2}^{1 - \beta } }}\left( {\frac{\partial }{{\partial x_{2} }}} \right)^{\beta } \frac{{{\Gamma }\left( {2 - \beta } \right)}}{{x_{2}^{1 - \beta } }}\frac{{\partial^{\beta } u_{1} }}{{\left( {\partial x_{2} } \right)^{\beta } }}$$

with the same initial and boundary conditions.

**Figure 4:**
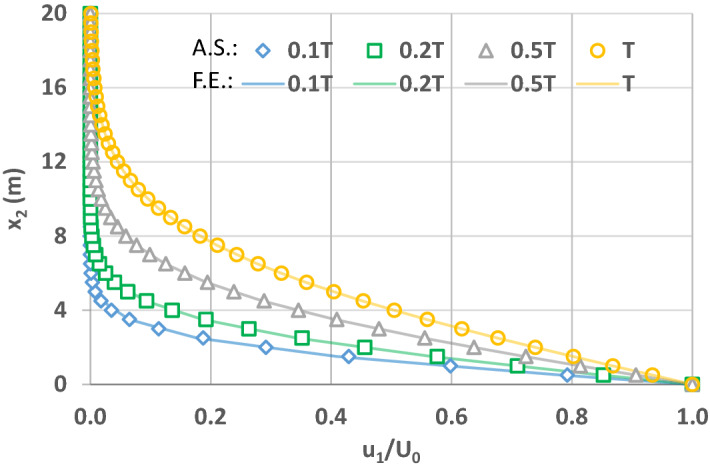
Velocity profiles of x_1_ direction velocity u_1_ normalized by boundary velocity U_0_ when space and time fractional derivative powers are 1. Solid lines correspond to simulations by fractional governing equation (F.E.) when time t = 0.1 T, 0.2 T, 0.5 T¸ T. Shapes (diamond, square, triangle, and circle) represent the corresponding analytical solutions (A.S.) of the conventional governing equation, respectively.

In order to solve Eq. (50) numerically, a first-order approximation of the Caputo’s fractional time derivative^45^ and a second-order accurate Caputo’s fractional space derivative^56^ schemes are coupled similar to the numerical solution of the fractional open channel flow problem as reported in Ercan and Kavvas^57^. When the fractional powers of space and time derivatives of Eq. (50) become one, the solution should converge to that of the conventional form of this Eq. (47). x_1_-direction velocity u_1_ normalized by the boundary velocity U_0_ (i.e., u_1_/U_0_) is plotted in Fig. 4. As shown in Fig. 4, the velocity profiles of the analytic solution (Eq. 49) of the conventional governing Eq. (47) compare well with those of the numerical solution of the fractional governing Eq. (50) when powers of the fractional space and time derivatives are one (β = α = 1).

**Figure 5:**
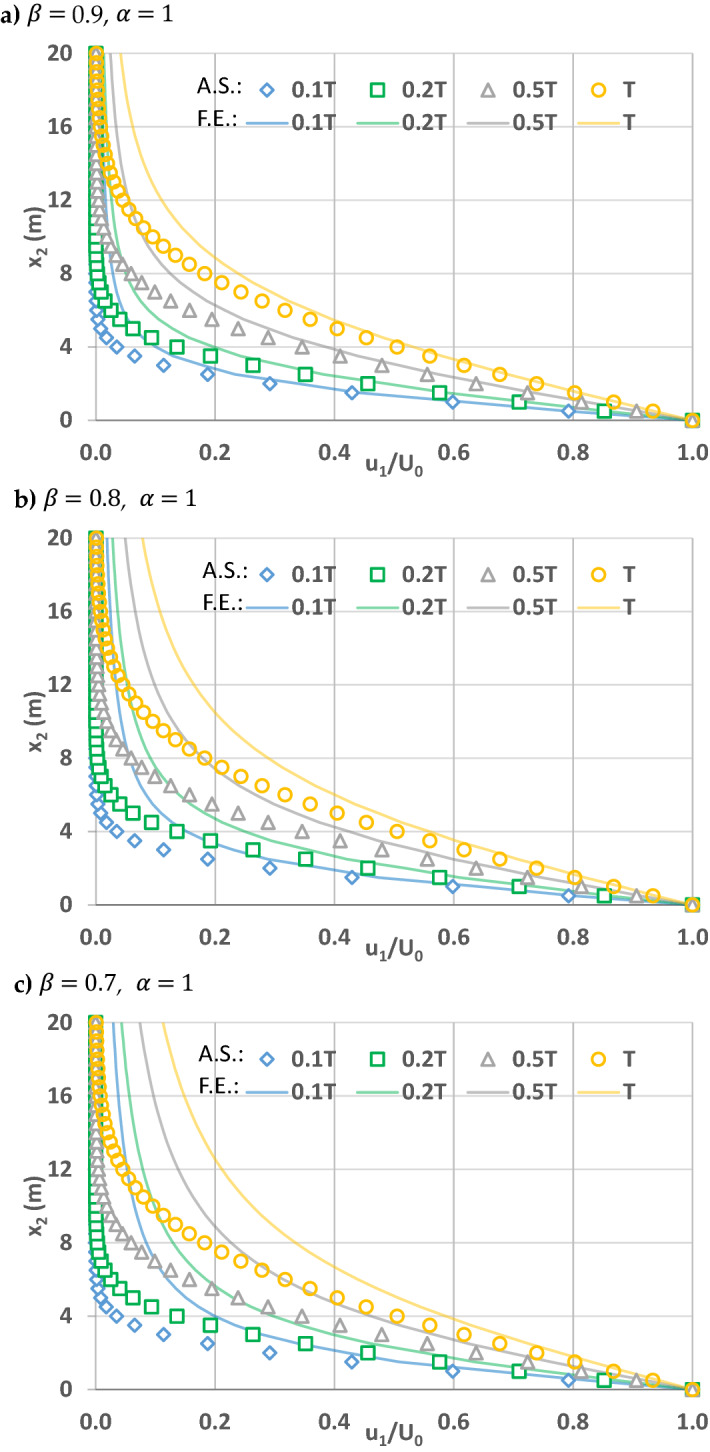
Velocity profiles of x_1_ direction velocity u1 normalized by boundary velocity U_0_ when time and space fractional derivative powers $\alpha =$ 1 and (**a**) $\beta =$ 0.9, (**b**) $\beta =$ 0.8, (**c**) $\beta =$ 0.7 $.$ Solid lines correspond to simulations by the fractional governing equation (F.E.) when time t = 0.1 T, 0.2 T, 0.5 T¸ T. Shapes (diamond, square, triangle, and circle) represent the analytical solutions (A.S.) of the conventional governing equation at corresponding times.

**Figure 6:**
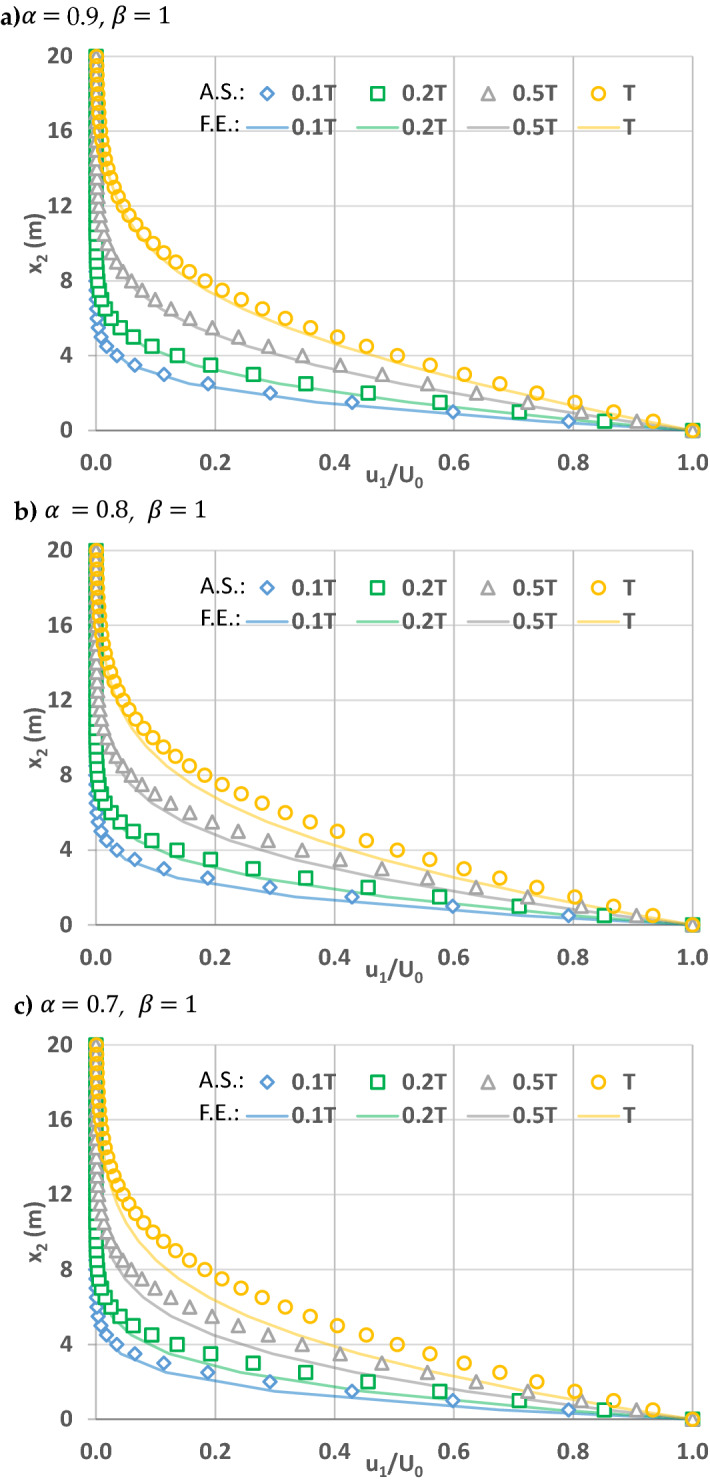
Velocity profiles of x_1_ direction velocity u1 normalized by boundary velocity U_0_ when space and time fractional derivative powers when $\beta = 1$ and (**a**) $\alpha =$ 0.9, (**b**) $\alpha =$ 0.8, (**c**) $\alpha =$ 0.7. Solid lines correspond to simulations by the fractional governing equation (F.E.) when time t = 0.1 T, 0.2 T, 0.5 T¸ T. Shapes (diamond, square, triangle, and circle) represent the analytical solutions (A.S.) of the conventional governing equation at corresponding times.

**Figure 7:**
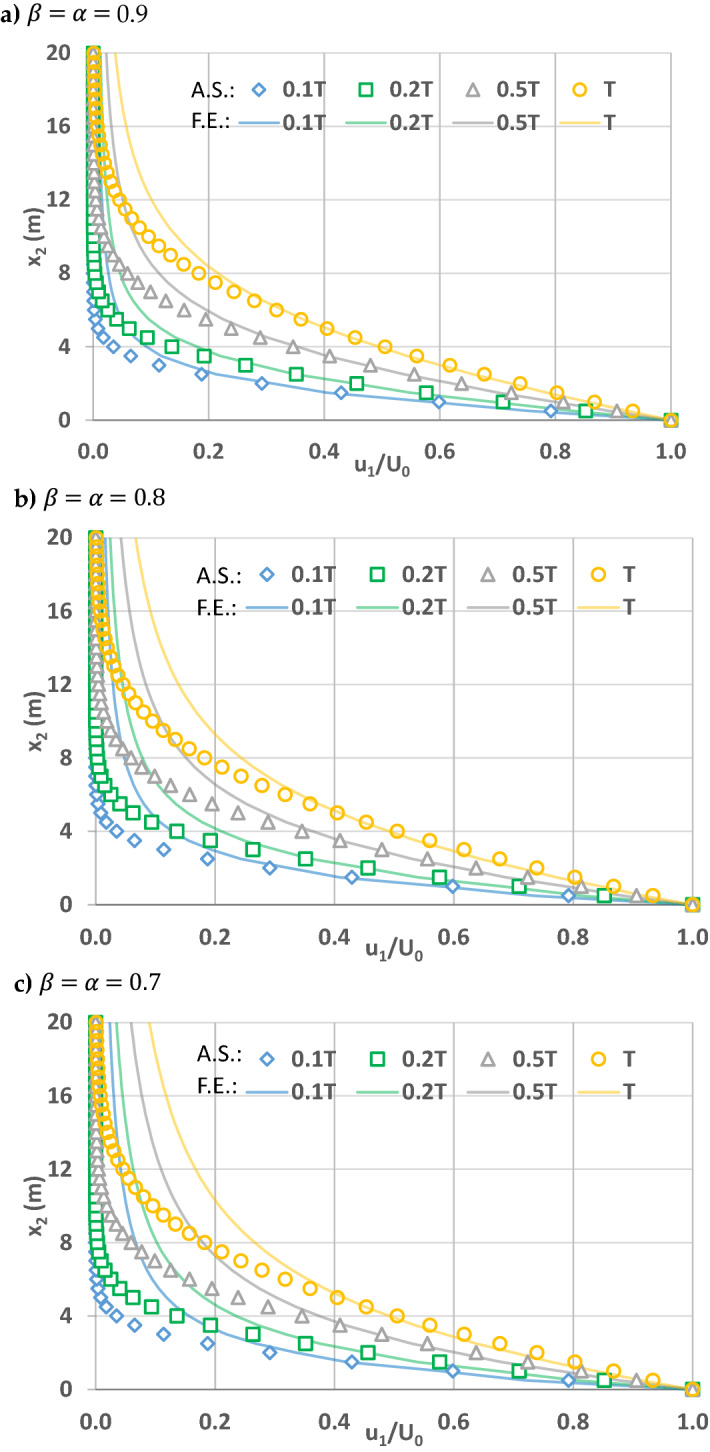
Velocity profiles of x_1_ direction velocity u1 normalized by boundary velocity U_0_ when space and time fractional derivative powers are equal, (**a**) $\alpha = \beta =$ 0.9, (**b**) $\alpha = \beta =$ 0.8, (**c**) $\alpha = \beta =$ 0.7. Solid lines correspond to simulations by the fractional governing equation (F.E.) when time t = 0.1 T, 0.2 T, 0.5 T¸ T. Shapes (diamond, square, triangle, and circle) represent the analytical solutions (A.S.) of the conventional governing equation at corresponding times.

In order to explore the contribution of powers of space and time fractional derivatives on flow, Figs. 5, 6 and 7 are plotted for the simple flow due to a wall suddenly set into motion. This is still a relatively simple flow problem due to above listed assumptions, and will help us to understand the effects of fractional powers on flow. As explained in Zaslavsky^30^, fractional governing equations account for the non-Fickian flow processes, including sub-diffusive (i.e., slow) and super-diffusive (i.e., fast) processes. In this regard, Zaslavsky^30^ defined the transport exponent of his fractional kinetic equation as µ = α/β, expressed here as such in order to be consistent with the above notation, to quantify competing time and space fractional derivative powers. Therefore, the transport exponent µ quantifies competing sub- and super-diffusivity. µ = 1 corresponds to normal diffusion, µ < 1 sub-diffusion, and µ > 1 super-diffusion. Before investigating the effects of the time and space fractional powers, note that flow velocities are fixed at the boundary conditions at $x_{2} = 0$ and $x_{2} = \infty$. Therefore, the effects are smaller in the vicinity of these boundaries and higher away from them. As such, the comparisons below for the solutions of fractional and integer order governing equations correspond to $x_{2}$ locations away from the boundaries.

For the case when the power of the time fractional derivative is one (α = 1), the velocities by fractional governing equations are higher as power of the space fractional derivative is less than one (β < 1). In this case Zaslavsky^30^’s transport exponent µ = α/β > 1 corresponds to super-diffusion. The difference between the velocity by the conventional governing equation (β = α = 1) and that by the fractional governing equation (when β < 1 and α = 1) increases through time for a fixed x_2_ location. The same initial condition is used for the fractional and integer governing equations; therefore, the velocity differences are smaller close to t = 0. Furthermore, for a fixed simulation time, the velocity for a specific x_2_ location (especially away from the moving plate) increases as the fractional space derivative power decreases from 1 (see Fig. 5a–c).

For the case when the power of the space fractional derivative is one (β = 1), the velocities by fractional governing equations are lower as the power of the time fractional derivative is less than one (α < 1). In this case Zaslavsky^30^’s transport exponent µ = α/β < 1 corresponds to sub-diffusion. The difference between the velocity by the conventional governing equation (or the fractional governing equation when β = α = 1) and that by the fractional governing equation (β < 1, α = 1) increases at later times because the initial condition dominates for smaller times, similar to α = 1 case. However, for a fixed simulation time, the velocity at a specific x_2_ location (especially away from the moving plate) decreases as the fractional time derivative power decreases from 1 (see Fig. 6a–c).

When the time and space fractional derivative powers are equal and less than one (β = α < 1), the effects of sub- and super-diffusion are superimposed (Fig. 7). The transport coefficient for this case becomes µ = α/β = 1, displaying normal diffusion in theory. However, as shown in Fig. 7, when α and β are equal and close to 1, the velocity, at a fixed x_2_ location close to the moving plate at $x_{2} = 0,$ does not change for both the conventional and the fractional governing equations. However, for a fixed simulation time, the velocity for a specific x_2_ location away from the moving plate increases as the fractional space and time derivative powers decrease from 1 (see Fig. 7a–c).

## Concluding remarks

The governing equations of unsteady multi-dimensional incompressible and compressible flows in fractional time and multi-fractional space were developed in this study. Since the time behaviour of fluid flows in various fields of engineering are as important as their behaviour in space, the governing multi-dimensional fluid flow equations not only in multi-fractional space but also in fractional time were attempted to be developed in this study. Also, due to the observed anisotropy in fluid flow processes in nature, the fractional scaling of fluid flows was addressed by different fractional powers in different Euclidean directions, resulting in governing equations in multi-fractional space. When their fractional powers in time and in multi-fractional space are specified to unit integer values, the developed fractional equations of continuity and momentum for incompressible and compressible fluid flows reduce to the corresponding conventional Navier–Stokes equations. As such, these fractional governing equations for incompressible and compressible fluid flows may be interpreted as generalizations of the conventional Navier–Stokes equations. For the frictionless flow conditions, the corresponding fractional governing equations were also developed as a special case of the fractional governing equations of incompressible flow. When their derivative fractional powers are specified to unit integers, these equations are shown to reduce to the conventional Euler equations.

The capabilities of the developed fractional governing equations of hydrodynamics were investigated by the first Stokes Problem (i.e., flow due to a wall suddenly set into motion). It was first shown that the results of fractional governing equations when powers of the fractional space and time derivatives are one (β = α = 1) are the same as the analytical solution of the conventional integer order governing equations. Next, the sub- and super-diffusive behaviour of the fractional governing equations were explained in the context of Zaslavsky^30^’s transport exponent framework. As shown in this numerical application’s results, the developed fractional equations of fluid flow have the potential to accommodate both the sub-diffusive and the super-diffusive flow conditions in addition to the conventional case.

## Data Availability

All data generated or analyzed during this study are available from the corresponding author upon reasonable request.
